# Variation in Thyroid-Stimulating Hormone and Cognitive Disorders in Unmedicated Middle-Aged Patients with Major Depressive Disorder: A Proton Magnetic Resonance Spectroscopy Study

**DOI:** 10.1155/2022/1623478

**Published:** 2022-09-05

**Authors:** Hui Zhao, Shunkai Lai, Shuming Zhong, Yiliang Zhang, Hui Yang, Yanbin Jia

**Affiliations:** ^1^Department of Psychiatry, First Affiliated Hospital of Jinan University, Guangzhou, 510630 Guangdong, China; ^2^Division of Sleep Medicine, Ganzhou People's Hospital, Ganzhou, 341099 Jiangxi, China; ^3^Department of Pathology, Ganzhou People's Hospital, Ganzhou, 341099 Jiangxi, China

## Abstract

**Background:**

Middle-aged (45-59 years old) patients with major depressive disorder (MDD) have a predilection for dementia and cognitive disorders (CDs); however, the characteristics and mechanisms of CDs in these patients remain unclear. There are also known connections between thyroid-stimulating hormone (TSH), brain biochemical metabolism, and cognitive function (CF); however, there is scanty of information about these connections in middle-aged MDD patients.

**Methods:**

Cognitive assessment was performed on 30 first-episode, untreated middle-aged patients with MDD and 30 well-matched healthy controls (HCs) using the MATRICS Consensus Cognitive Battery (MCCB). N-acetyl aspartate (NAA)/creatine (Cr) and choline (Cho)/Cr ratios in the prefrontal cortex (PFC) and cerebellum were also obtained via proton magnetic resonance spectroscopy (^1^H-MRS), and the TSH level was measured by chemiluminescence analysis.

**Results:**

MDD patients presented significantly lower processing speed, working memory, verbal learning, reasoning problem-solving, visual learning, and composite cognition scores than controls, with a statistically lower NAA/Cr ratio in the right cerebellum. Age was positively related to reasoning problem-solving in the MDD group (*r* = 0.6249, *p* = 0.0220). Education also showed a positive association with visual learning, social cognition, and composite cognition. The 24-item Hamilton Depression Rating Scale (HDRS-24) score was negatively related to all domains of CF. TSH levels were markedly decreased in the MDD group, and a positive connection was determined between the NAA/Cr ratio in the right PFC and the TSH level.

**Conclusions:**

Middle-aged MDD patients have multidimensional CDs. There are changes in PFC and cerebellar biochemical metabolism in middle-aged patients with MDD, which may be related to CDs or altered TSH levels.

## 1. Introduction

The World Health Organization projects that major depressive disorder (MDD), a condition characterized by low mood, loss of pleasure, and/or suicidal ideation [1], will present the largest source of the global burden of disease by 2030. Middle age is an important developmental period between youth and old age, and individuals at this stage of life face enormous pressure and an increased risk of depression. Unfortunately, most of current depression-associated research focuses on patients in childhood, adolescence, and early adulthood, with few studies investigating middle-aged depressive patients. Nevertheless, depression in middle-aged adults has become an important public health issue, predisposing these patients to the development of dementia and cognitive disorders (CDs). The characteristics and mechanisms underlying such CDs, however, also remain to be elucidated.

CDs belong to a well-recognized feature of MDD that can persist even during disease recovery [2]. There is accumulating evidence that patients with depression have impairments in working memory (WM) [3], processing speed [4], and executive function [5]. Persistent cognitive symptoms can seriously impair the quality of life of depressive patients; therefore, it is of vital importance to understand and assess these symptoms. Defining the role of CDs in the psychopathology and treatment of MDD, however, remains challenging. Past literature generally centered on specific components of cognitive function (CF) without taking a more comprehensive and systematic approach. Accordingly, there is an urgent need to study the characteristics of CDs in middle-aged patients and its associated neural mechanisms in patients with middle-aged depression.

Neuroimaging sheds new light on the pathogenesis of MDD and CDs. In first-episode MDD patients, the volume of the dorsal medial prefrontal cortex (PFC) has been shown to be reduced, suggesting that MDD may be related to structural changes in this region [6]. The cerebellum also plays a vital part in the modulation of cognition and emotion. A study by Lai et al. showed that cerebellar gray matter reduction is strongly associated with the pathophysiology of MDD patients [7]. In addition, cerebellar damage has been shown to lead to impairments in spatial memory, executive function, and emotional regulation [8]. Alterations in the structural and functional connections of the PFC and cerebellum are strongly linked to the occurrence of MDD and CDs; thus, understanding these key brain regions is critical for elucidating the mechanisms underlying these disease processes.

Previous studies have indicated potential associations between endocrine disturbances and MDD [9]. In addition, hypothalamic-pituitary-thyroid (HPT) axis abnormalities have been demonstrated to be critical in MDD pathophysiology and treatment [10]. Previous evidence has also documented that MDD patients have reduced thyroid-stimulating hormone (TSH) levels than healthy controls (HCs) [11]. In addition, TSH has been shown to be essential for regulating neuronal differentiation and synaptic plasticity. With improvements in imaging techniques, multiple studies have documented that TSH modulates various cerebral functions involved in cognition and mood. However, there have been no studies related to the role of TSH in middle-aged depressed patients. And it remains to be clarified if TSH levels change or correlate with brain biochemical metabolism and CF in such patients.

Combining evaluations of metabolic brain alterations, TSH levels, and neurophysiological test results may therefore be an effective method to explore the pathogenesis of depression. In this study, we propose a hypothesis of possible changes in PFC and cerebellar biochemical metabolism in middle-aged patients with MDD, which may relate to CDs or changes in TSH levels. The novelty and motivation of this research was to elucidate the neural mechanisms of CDs in middle-aged depressive patients, so as to provide more effective treatment for such patients.

## 2. Materials and Methods

### 2.1. Participants

This study included 30 patients (10 males, 20 females; mean ± SD: 50.85 ± 4.69 years of age) who had been diagnosed with MDD by two experienced clinical psychiatrists (Yanbin Jia and Shuming Zhong, with 25 and 6 years of clinical psychiatry experience, respectively) based on the *Diagnostic and Statistical Manual of Mental Disorders*, 4th edition (DSM-IV) [12]. In addition, 30 HCs, consisting of 13 males and 17 females with a mean age of 50.18 ± 5.19, were enrolled. All the 60 subjects included in our study were native Han and right-handed, and underwent baseline MRI within 2 days since the first contact. This study was approved by the Ethics Committee of the First Affiliated Hospital of Jinan University as an exempt study without a registration number. Due to the retrospective nature of the study, no clinical registration was required. These subjects volunteered to participate after a full written and verbal explanation of the study, with informed consent provided.

### 2.2. MDD Group

Thirty MDD adults diagnosed between May 2017 and May 2020 were recruited from the outpatient and inpatient psychiatric departments of our hospital. All patients were experiencing their first attack of MDD and were drug-naive. The enrolled MDD patients all met the following criteria: (i) meeting the DSM-IV criteria for first-episode, current, and unipolar MDD; (ii) age range: 45-59 years; (iii) 24-item Hamilton Depression Rating Scale (HDRS-24) score ≥ 20 [13]; (iv) Young Mania Rating Scale (YMRS) score < 6 [14]; and (v) educational level > junior high school.

In contrast, patients in accordance with any of the following were excluded: (i) axis I or II psychiatric disorders; (ii) pregnant, lactating, or postpartum women; (iii) previous cerebrovascular diseases (cerebral infarction, hemorrhage, tumor, etc.), organic diseases of the brain, or head injuries; (iv) history of alcohol, illicit drug abuse, substance use disorders, or substance addiction; (v) history of neurological disorders or magnetic resonance contraindications (braces, metallic implants, or claustrophobia); (vi) previous psychotropic medication use or electroplexy; or (vii) severe vision or hearing impairment.

### 2.3. HC Group

Additional HCs (13 males and 17 females) matched for age, gender, and educational level with MDD cases were enrolled. These volunteers were recruited via local advertisements and posted flyers and were carefully screened by the psychiatrists who participated in the study to exclude any current or prior psychiatric illnesses. Participants in the HC group were recruited based on the following criteria: (i) healthy adult; (ii) age range: 45 to 59; (iii) HDRS-24 score < 8; (iv) YMRS score < 6; and (v) educational level > junior high school. The exclusion criteria for the MDD group also applied to the HC group.

### 2.4. Assessment

The following clinical information was recorded for all subjects at their first visit: age, sex, education background, and occupation. The HDRS-24, a widely used depressive symptom severity assessment tool with good reliability, was used to assess participants' depression symptoms. A subject was defined as depressed if the total score was between 20 and 35, and severe depression was indicated if the total score was over 35. While a score < 8 was considered normal. The YMRS was used to assess manic symptoms in subjects. This tool has been widely used in the evaluation of mania severity in clinical trials with good interrater reliability, validity, and sensitivity. Subjects were considered manic if their total score was over 20. If the total score was below 6, it was considered normal.

Participants' CF was assessed using the MATRICS Consensus Cognitive Battery (MCCB) [15]. Initially, the MCCB was developed for patients with schizophrenia; however, in recent years, it has also been adopted for CF assessment of patients with bipolar disorder [16] and MDD [17, 18]. There is also Chinese simplified MCCB, and co-norming and standardization have been performed in China with good test-retest reliability [19]. This instrument assesses seven cognitive-associated dimensions using 10 subscales, including (i) processing speed through the following three subtests: Trail Making Test-A (TMT-A) [20], Category Fluency (Animal Naming), and the Brief Assessment of Cognition in Schizophrenia-Symbol Coding (BACS-SC); (ii) attention/vigilance through the Continuous Performance Test-Identical Paris (CPT-IP); (iii) WM through the Wechsler Memory Scale-Third Edition Spatial Span (WMS-III SS); (iv) verbal learning through the Hopkins Verbal Learning Test-Revised (HVLT-R); (v) visual learning through the Brief Visuospatial Memory Test-Moderated (BVMT-R); (vi) reasoning and problem-solving ability through the Neuropsychological Assessment Battery: Maze (NABM); and (vii) social cognition through the Mayer-Salovey-Caruso Emotional Intelligence Test. Higher MCCB test scores indicated better CF.

### 2.5. Image Data Acquisition

Changes in PFC and cerebellar metabolism were observed by ^1^H-MRS. In all subjects, MRI data were obtained at the Radiology Department of our hospital using a Discovery MR750 3.0-T System (General Electric, Milwaukee, WI, USA) equipped with an 8-channel head coil array. Participants lay supine during imaging, with foam padding and earplugs utilized to reduce head motion and scanner noise during the scan. They were guided to relax and keep their eyes closed and their heads still while scanning. First, a routine cranial MRI sequence was performed to exclude brain lesions. Routine axial *T*_1_-weighted (*T*_1_W) fluid attenuation inversion recovery (*T*_1_ Flair) (repetition time [TR] = 1800 ms, echo time [TE] = 24 ms) and fast-spin echo *T*_2_W MR images (TR = 4500 ms, TE = 120 ms) were obtained. Two experienced radiologists confirmed no cerebral signal or structural abnormalities.

**Figure 1:**
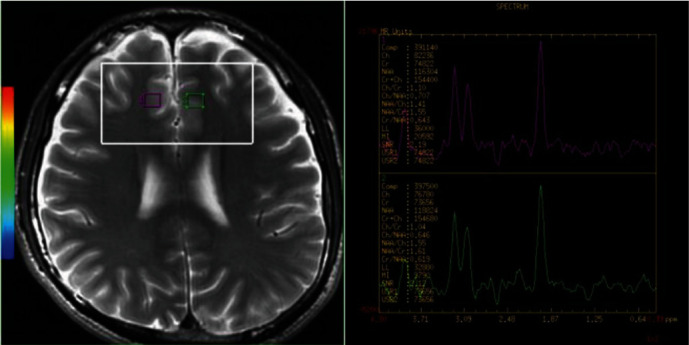
MRI showing the location of MRS of a VOI placed in the prefrontal cortex, with ^1^H-MRS in the bilateral prefrontal cortex. The large white box shows the VOIx for MRS acquisition, and the small ones depict individual VOIs for spectral analysis. MRI: magnetic resonance imaging; MRS: magnetic resonance spectroscopy; VOI: volume of interest; ^1^H-MRS: proton magnetic resonance spectroscopy; NAA: N-acetylaspartate; Cho: choline; Cr: creatine.

**Figure 2:**
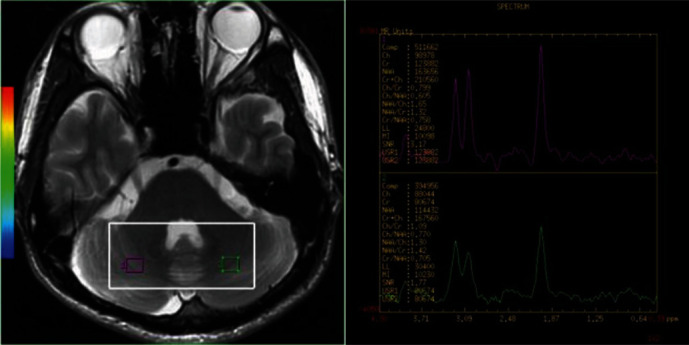
MRI showing the location of MRS of the VOI placed in the cerebellum, with ^1^H-MRS in the bilateral cerebellum. The large white box shows the VOIx for MRS acquisition, and the small ones depict individual VOIs for spectral analysis. MRI: magnetic resonance imaging; MRS: magnetic resonance spectroscopy; VOI: volume of interest; ^1^H-MRS: proton magnetic resonance spectroscopy; NAA: N-acetylaspartate; Cho: choline; Cr: creatine.

**Figure 3:**
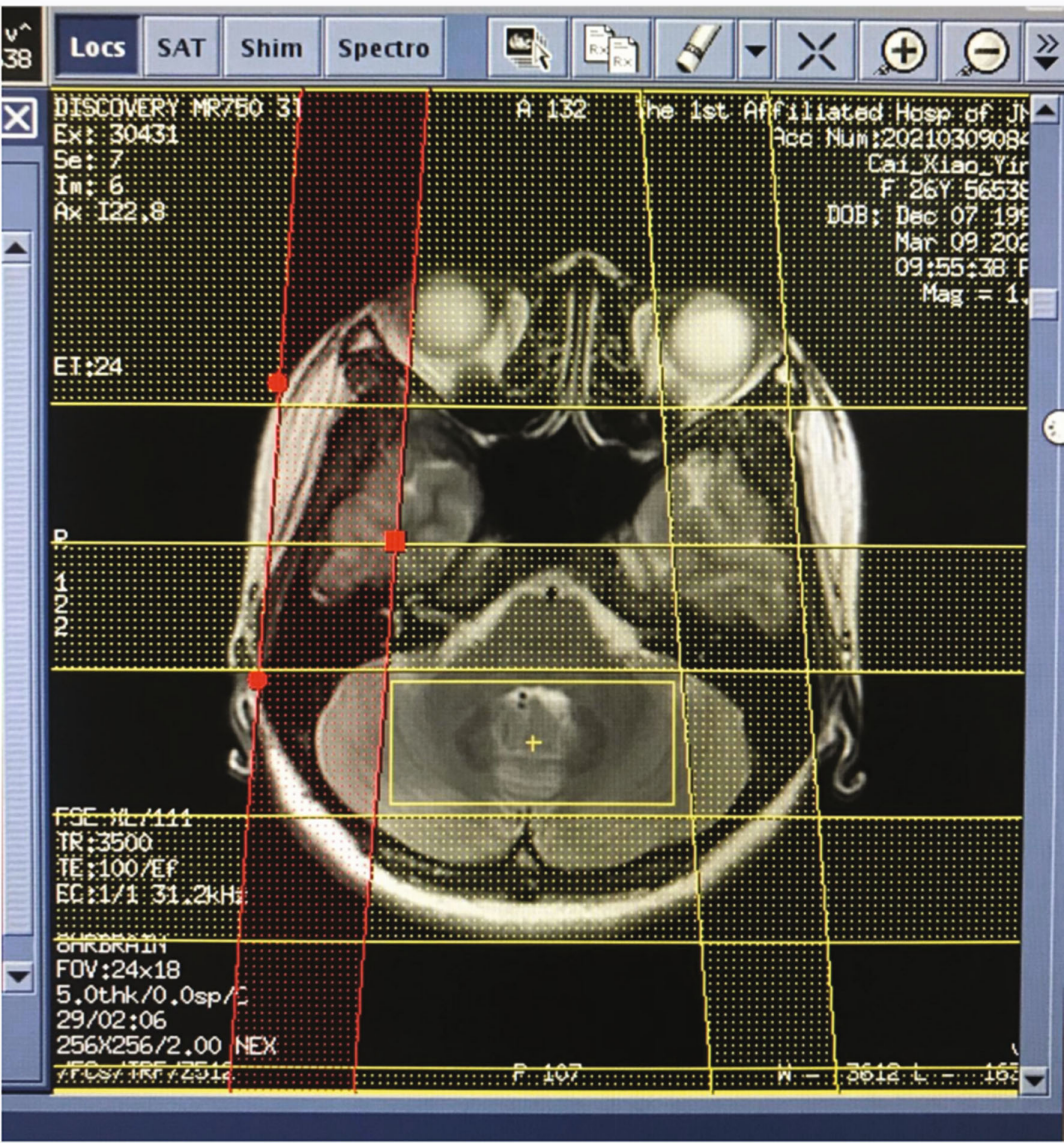
“Saturation bands” location.

Anatomical localization (TR = 5000 ms, TE = 113 ms, slice thickness = 5 mm with no gap) was performed using axial *T*_2_W MR images. The volume of interest (VOI), including 55 voxels with the nominal dimension set as 7.5 × 7.5 × 10 mm^3^, was uniformly positioned by the same investigator. Figures 1 and 2 show the VOI locations on bilateral PFC and cerebellar scans of the brain, respectively. For the acquisition of two-dimensional (2D) multivoxel ^1^H-MRS, a point resolved spectroscopy sequence (PRESS) was used, with a chemical shift selective saturation (CHESS) pulse applied for water suppression. The influence of peripheral tissues on scanning results was minimized using the saturated zone. Scan parameters are as follows: TR = 2000 ms, TE = 32 ms, slice thickness = 15 mm, numbers of excitation = 1, matrix = 16 × 16, and field of view = 10 × 10 cm. Outside the VOI, extra saturation bands (Figure 3) were placed to reduce lipid contamination from the scalp. Shimming of magnetic homogeneity was done before the spectroscopic acquisitions. A 7-9 Hz line width (full width at half maximum, FWHM) was usually achieved on the water resonance of the VOI. Universal quality standard spectra with a FWHM > 10 Hz or water suppression > 98% were excluded. The total ^1^H-MRS sequence acquisition time was 5 min 28 s.

Spectral datasets were analyzed using the manufacturer-supplied software package program of the MR system (GE Advantage Workstation: AW4.2_07). Voxels were relocalized to predefined brain regions, namely, the left and right PFC (Figure 1) and the cerebellum (Figure 2). N-acetyl aspartate (NAA), choline (Cho), and creatine (Cr) were the metabolites measured, with their chemical shifts being 2.02 ppm, 3.22 ppm, and 3.02 ppm, respectively. “Cho” is composed of phosphocholine (PCho) plus glycerophosphocholine (GPCho) and choline is below the detection limit. “Cr” is technically the summation of creatine (Cr) plus phosphocreatine (PCr). NAA/Cr and Cho/Cr ratios were calculated. Voxel placements for spectroscopy as well as all data were analyzed by a well-trained radiologist with no knowledge of the diagnosis of each participant.

### 2.6. Assessment of Serum TSH Levels

Blood (5 mL) samples, routinely collected by venipuncture during the morning hours within 24 hours after MRI, were obtained from all participants for TSH detection. The direct chemiluminescence method was used to determine TSH concentrations. The same assay, run per individual, was performed to examine the samples. Normal thyroid function was indicated if serum TSH concentration was within the range of 0.49–4.91 mIU/L.

### 2.7. Statistical Analyses

Statistical analyses were carried out with SPSS v23.0, and two-tailed significance was set at *p* value < 0.05. First, assessment of data distribution normality used the Kolmogorov-Smirnov goodness-of-fit test. For intergroup comparisons of demographic information, baseline data, CF performance, and biochemical metabolite ratios, the *t*-test and the Mann-Whitney *U* test were employed for normally distributed data and nonnormally distributed data, respectively. As to multiple comparisons of metabolite concentrations, the false discovery rate (FDR) procedure was used for alpha-level correction, with the postcorrection (*p*_adj_) significance level set at 0.05. Gender distribution was analyzed using the chi-square test. Second, correlation analyses were carried out to examine the correlations between CF, brain biochemical metabolite ratios (NAA/Cr, Cho/Cr), and TSH levels in the MDD group. To analyze the correlation between CF and biochemical metabolite ratios in the PFC and cerebellum, confounding variables (clinical variables: the years of education, the HDRS-24 score and age) were controlled. If two sets of data were normally distributed, the Pearson's correlation test was used; otherwise, the Spearman's correlation test was adopted. The equation of linear regression and graphs were performed with GraphPad Prism 8.0 (GraphPad Software Inc., La Jolla, CA, USA).

## 3. Results

### 3.1. Demographic Information, Clinical Data, and TSH Levels

See Table 1 for the demographic and clinical data of both cohorts of subjects that were matched for age (*Z* = −0.781, *p* = 0.435), sex (*X*^2^ = 0.334, *p* = 0.563), YMRS score (*Z* = −0.654, *p* = 0.513), and education background (*Z* = 0.628, *p* = 0.530). Age, gender, or education background differed insignificantly between cases and controls. The two groups did, however, have significantly different HDRS-24 scores (*Z* = −6.377, *p* = 0.001) and TSH levels (*Z* = 3.591, *p* = 0.001).

### 3.2. CF Analysis in the MDD and HC Groups

The CF performance of both groups assessed by the MCCB test is shown in Table 2. The HC group showed statistically higher scores in the dimensions of processing speed (*t* = −4.248, *p* = 0.001), WM (*t* = −2.566, *p* = 0.021), verbal learning (*t* = −2.302, *p* = 0.033), visual learning (*t* = −3.309, *p* = 0.004), reasoning problem-solving ability (*t* = −3.193, *p* = 0.004), and composite cognition (*t* = −4.494, *p* = 0.001) than the MDD group. While no obvious differences were found in attention/vigilance (*t* = −2.004, *p* = 0.058) and social cognition (*Z* = −1.772, *p* = 0.076).

### 3.3. Comparison of Bilateral PFC and Cerebellum Biochemical Metabolite Ratios (NAA/Cr and Cho/Cr)

As shown in Table 3, a lower right cerebellum NAA/Cr (*t* = −3.015, *p* = 0.032) was determined in the MDD group compared with the HC group, but no statistically significant differences were observed in other biochemical metabolite ratios.

### 3.4. Correlations between CF and Clinical Data in MDD Group

**Figure 4:**
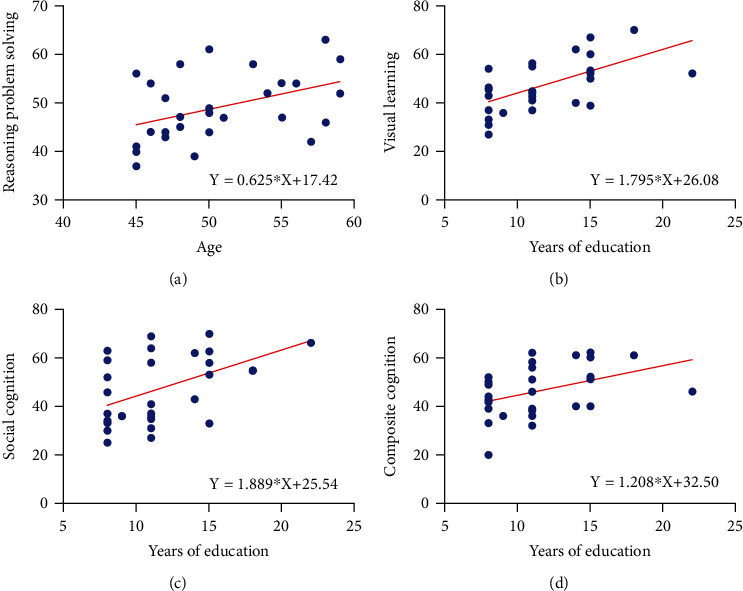
Correlations of cognitive function with patient baseline data in MDD group. (a) The correlation between reasoning problem-solving and age. (b) The correlation between visual learning and years of education. (c) The correlation between social cognition and years of education. (d) The correlation between composite cognition and years of education.

We also analyzed the correlations between CF and clinical data in the MDD group (Figure 4). Age (*X*) was positively related to reasoning problem-solving (*Y*) in the MDD group. The equation of linear regression is *Y* = 0.625∗*X* + 17.42 (*p* = 0.022). Education (*X*) also showed a positive association with visual learning (*Y*), social cognition (*Y*), and composite cognition (*Y*). The equations of linear regression are *Y* = 1.795∗*X* + 26.08 (*p* = 0.001), *Y* = 1.889∗*X* + 25.54 (*p* = 0.010), and *Y* = 1.208∗*X* + 32.50 (*p* = 0.027), respectively. The HDRS-24 score was negatively related to all domains of CF.

### 3.5. Correlations of CF with PFC and Cerebellum Biochemical Metabolite Ratios

The results determined no correlations of CF with PFC and cerebellum NAA/Cr and Cho/Cr ratios in the MDD group.

### 3.6. Correlations of Biochemical Metabolite Ratios with MDD Patients' Clinical Data

**Figure 5:**
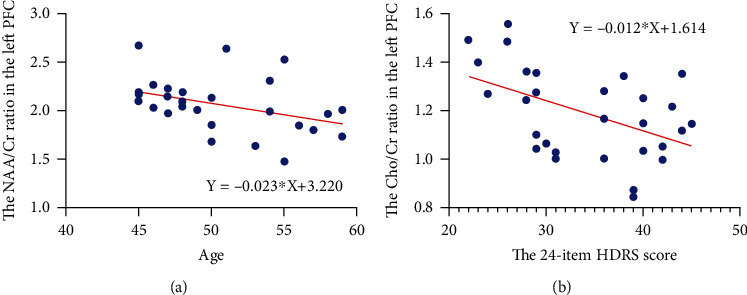
Correlations of biochemical metabolite ratios with clinical MDD data. (a) The correlation between left PFC NAA/Cr ratio and age. (b) The correlation between left PFC Cho/Cr ratio and 24-item HDRS score.

In the MDD group, the left PFC (*Y*) NAA/Cr ratio was inversely linked to age (*X*) (Figure 5(a)), with the equation of linear regression of *Y* = −0.023∗*X* + 3.220 (*p* = 0.035), while a negative association between the left PFC (*Y*) Cho/Cr ratio and the HDRS-24 score (*X*) was determined (Figure 5(b)), and the equation of linear regression is *Y* = −0.012∗*X* + 1.614 (*p* = 0.008). There were no correlations between biochemical metabolite ratios in the cerebellum and clinical data.

### 3.7. Correlations between Clinical Variables, Biochemical Metabolite Ratios, CF, and TSH Levels

**Figure 6:**
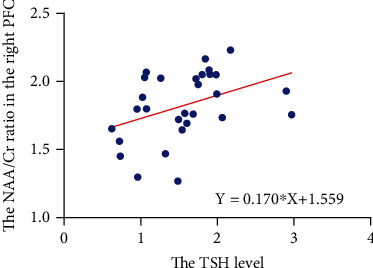
Correlations between clinical variables, biochemical metabolite ratios, cognitive function, and TSH levels.

For the MDD group, the right PFC (*Y*) NAA/Cr ratio was positively linked to the TSH level (*X*). The equation of linear regression is *Y* = 0.170∗*X* + 1.559 (*p* = 0.033) (Figure 6). No significant correlations were identified among clinical variables, CF, and TSH levels.

## 4. Discussion

This study, to our knowledge, was one of the first to determine the relationship between biochemical abnormalities, CF, and TSH levels using multivoxel ^1^H-MRS in first-episode, treatment-naïve, middle-aged patients with MDD.

### 4.1. Characterizing CDs in Middle-Aged MDD Patients

The paper took the initiative to evaluate the CF of middle-aged patients with MDD using the MCCB, so as to clarify the correlation of biochemical abnormalities with CF. We used the TMT-A, BACS-SC, and Category Fluency (Animal Naming) tests to obtain the level of processing speed. The results revealed obviously lower processing speed in MDD patients than in HCs, as has been demonstrated previously by other researchers [21]. Information processing has been shown to enable focus on goal-relevant information while ignoring goal-irrelevant information [22]. Processing speed is reported to be correlated with depressive symptom load [23], which is consistent with our results. However, there was no clear correspondence between the severity of depression and the degree of CDs, as there was no single linear relationship. It also remains unclear to what extent depressive symptoms develop into CDs.

This study used CPT-IP to detect attention/vigilance in middle-aged depressed patients. The selective attention response time of depressive patients has been indicated to be longer than a normal group [24], with a previous study indicating that patients with depression have slower response times to environmental stimuli and sustained impairments in attention [25]. However, the results of our study were not consistent with these previous findings, possibly due to age differences in the samples. Attention can be divided into selective and sustained attention, each focusing on different facets of this process; therefore, the results obtained may be different depending on the type of attention tested. Most existing studies only measure a part of the attention module. In the future, it would be useful to use various cognitive tests to provide a more comprehensive evaluation of attention, so as to explore whether there are differences in attention modules and whether attention deficits influence daily life function, treatment, and rehabilitation of depressive patients.

WM, which is defined as the ability to memorize, retrieve, and utilize information for a limited period of time, underlies multiple cognitive tasks and day-to-day activities [26]. WM plays a vital part in cognitive control, preventing self-regulatory goals from being interfered with by desire-related thoughts and emotions [27]. Severely impaired WM is common in MDD [25]. In the present study, it was somewhat unexpected to find no significant differences in WM capacity between different age groups of patients with depression. Differences in clinical features of subjects, including medication or first-episode versus recurrent depression, may have contributed to these inconsistent results. Depression severity has been shown to have a significant inverse association with measures of WM [28]. They are less sensitive to rewards and positive effects, which will lead to the appearance of biases and negative information.

We used the HVLT-R to judge verbal learning performance in middle-aged depressive patients and found their problems with verbal learning, which is similar to what has been reported in the studies of Rund et al. and Bora et al. [29, 30]. The late-life depression group has also been shown to have greater impairment in verbal learning [31]; however, this evaluation was carried out using different, though related, tests. Depression is now widely believed to be linked to episodic memory and learning deficits.

Visual learning mainly refers to the process by which the visual organs recognize information and then store and process them in the brain. In this study, a short visual spatial memory test was used to measure the visual learning ability of middle-aged patients with depression, and impaired visual learning was found in these patients. Visual learning can be divided into two types, namely, immediate memory and delayed memory. Some researchers believe that both types are impaired in depression [32]; however, others believe that delayed-memory visual learning, other than the immediate-memory visual learning, is significantly worsened in patients with depression [33, 34]. It is widely recognized that impaired visual learning is one of the core symptoms of depression. Future research should reexamine visual learning ability based on the severity of depression in greater depth.

Reasoning problem-solving is the main component of executive function, which is the ability to plan, sort, and monitor one's behaviors. Fossati et al. believe that CDs in depression have the most significant impact on executive function [35]. The severity of executive function impairment has been confirmed to be positively linked to depressive symptom severity [36], which is also consistent with our research. Executive dysfunction develops as depression progresses; however, it is not fully recovered during the remission period, even after relief of depression symptoms. Executive function may also be an indicator of the severity of depression.

It is known that social cognition impairment may lead to poor social functioning [37]. A hallmark of MDD is significant social functioning impairment. Social cognition is defined as mental activities that enable others to understand their thoughts and feelings, a process that is essential in interpersonal relationships. MDD patients tend to interpret neutral expressions more negatively than normal controls [38]. Besides, there is a cognitive bias in the recognition of sadness and fear [39]. We found a correlation between social cognition and education in patients with depression, with a higher level of education being protective for social cognition. Some studies suggest that the impacts of age on CF are related to the regulation of cognition through dopamine neurotransmission. The effectiveness of dopamine neurotransmission decreases with age. However, we failed to demonstrate this in our subjects due to a narrow age range, and the effectiveness of dopamine neurotransmission was maintained at a stable level.

We found that social cognition, composite cognition, and visual function of middle-aged patients with depression were positively related to their education level. Recent foreign studies have shown a close association between early education and cognitive ability in later years. Also, a lower education level is considered as a predictor of CDs in later years [40]. Education level and cultural background affect not only concept formation and language performance but also CF such as visual spatial structure, visual perception ability, and memory [41], similar to our findings. Therefore, educational factors play a very important part in cognitive ability.

### 4.2. Biochemical Metabolite Ratios of PFC and Cerebellum

The present study used the ^1^H-MRS technique to evaluate whether middle-aged MDD patients had metabolic changes in the PFC and/or cerebellum. The MDD group was found to have evidently lower right cerebellum NAA/Cr ratios than HCs, consistent with previous studies [42, 43]. NAA is mainly present in the mitochondria of neural cells and is almost entirely stored in the neuronal cell body and nerve axon, which can therefore reflect nerve dysfunction [44]. This study found a lower NAA ratio that indicates reduced neuronal density in middle-aged MDD patients, suggesting neuronal dysfunction in such patients. Some studies also indicated no significant difference in the PFC NAA/Cr ratio between MDD patients and HCs [45]. However, some other research has reached different conclusions. These discrepant findings may be attributed to differences in age, disease duration, medication, and imaging data processing and analysis methods. We found that age was related to left PFC NAA/Cr values. The potential contribution of age to the metabolic effects on the PFC found in our study should be considered in future studies. In particular, a frontal lobe hypothesis of age-related cognitive changes has been proposed [46]. And our findings support this hypothesis.

Acetylcholine is a neurotransmitter closely related to emotion. Cho, one of the components of cell membrane phospholipid metabolism, is an indicator reflecting cell membrane transport function and cell proliferation [47]. A reduction in Cho may indicate intracellular signal transduction system impairment in MDD patients. According to our research results, middle-aged MDD patients had a markedly lower right cerebellum Cho/Cr ratio than HCs, suggesting the presence of abnormal cerebellum neural activity in early-stage depressive patients. There is great involvement of the intracellular signal transduction system impairment in the right cerebellar region in the pathophysiology of middle-aged MDD patients; however, the existing findings are controversial, with other studies finding an elevated cerebellum Cho/Cr ratio in these cases [48]. This inconsistency may result from differences in age, presence of comorbid conditions, disease course, and illness severity of participants in these studies.

### 4.3. Change in TSH Level

The connection between the brain and TSH levels in middle-aged patients with depression remains unclear. Our goal was to clarify any possible correlations between clinical presentations, TSH levels, and biochemical metabolite ratios in first-episode, untreated MDD patients, potentially contributing to useful information for nosogenesis underlying the disease and therapeutic strategies for such patients. TSH is involved in the body's metabolism and plays an important part in the development of the central nerve system, which can affect various neurologic functions, including behavior and sensation. Some studies have shown the connection between changes in TSH levels and depression [49]. The present study found obviously lower TSH levels in depressive patients than in HCs, suggesting that patients with depression may have dysfunction in TSH. Talaei et al. also found that low levels of TSH increased the prevalence of depression [50]. TSH, a sensitive indicator of thyroid hormone levels, can reflect mood changes, including depressive symptoms, as has been reported in some studies. The low TSH level in patients with depression may stem from several potential factors. First, 30-50% of depressive patients have hypothalamic-pituitary-adrenal (HPA) axis disorders, and increased glucocorticoid levels can result in decreased TSH secretion, with nighttime impairments in the TSH secretion peak affecting decreases in TSH levels [51]. Second, the hypothalamic-pituitary-thyroid axis (HPT) axis in these patients may exhibit dysfunction in negative-feedback regulation, whereby TSH release cannot be upregulated through negative feedback regulation; thus, these patients present with low concentrations of TSH. But some researchers indicated no notable difference in serum TSH levels between depressive patients and HCs, which could possibly be associated with a phenomenon known as “brain hypothyroidism” [48, 52]. Another possible reason for the inconsistent results is because of the different age ranges, as TSH secretion reduces with age. Nevertheless, to better understand this complex relationship, further research is warranted to examine the alterations of intracerebral and peripheral TSH levels in different populations.

We also found a positive correlation between the right PFC NAA/Cr ratio and TSH in middle-aged MDD patients. Many studies have shown changes in cerebral glucose metabolic rate and regional cerebral blood flow in patients with hypothyroidism [53] TSH level is also directly linked to brain metabolism [54]. Our study indicates that right PFC neuronal dysfunction may be correlated with abnormal TSH secretion in first-episode middle-aged MDD patients. Meanwhile, there were alterations in neuronal and thyroid function, possibly associated with the nosogenesis of depression. There has also been a study showing an association between TSH level and CF [55].

### 4.4. Correlations among Clinical Variables, TSH Levels, and Biochemical Metabolite Ratios

WM plays a key role in completing complex cognitive tasks. Austin et al. [56] found a correlation between WM and right PFC NAA/Cr values, indicating that prefrontal function and WM are closely related. In fact, brain imaging studies suggest that frontal loop abnormalities may be an important neuropathological mechanism for WM dysfunction in depressive patients [57, 58]. This study did not find a correlation between WM and biochemical metabolite ratios in the PFC. These inconsistent findings may be due to the combined effects of noncontrolled medication and aging on biochemical metabolites identified in previous reports. In this research, we exclude the impact of medication and aging by enrolling treatment-naive MDD patients alone, which ensured the high reliability of experimental results.

## 5. Conclusion

This paper preliminarily confirms the involvement of PFC and cerebellum in the development of MDD in middle-aged patients, providing novel insights into the nosogenesis of MDD and early prevention. In addition, neuronal dysfunction in the PFC may be linked to abnormal TSH levels in MDD, possibly due to the neuropathology of depression. In conclusion, the theoretical contribution of this study is to delineate the recognition of dysfunction in middle-aged patients with depression that can be used to clarify the pathophysiology of this disorder. There was, however, some room for improvement in this study. First, the sample size was relatively small and needs to be increased in the future. In addition, other metabolites (e.g., small molecules like glutamic acid and inositol) were not measured and may result in the omission of some useful information. Also, no follow-up investigations were conducted in this study, which should be supplemented in future research.

## Figures and Tables

**Table 1:** Demographic information, clinical data, and TSH level of subjects (mean ± SD).

	MDD group	HC group	*Z* or *X*^2^	*p* value
Age (years)	50.85 ± 4.69	50.18 ± 5.19	-0.781	0.435^a^
Gender (male/female)	10/20	13/17	0.334	0.563^b^
Education (years)	11.86 ± 3.56	12.05 ± 3.28	0.628	0.530^a^
HDRS-24 score	34.01 ± 7.10	3.56 ± 1.52	-6.377	0.001^a^^∗^
YMRS score	0.68 ± 0.75	0.60 ± 0.88	-0.654	0.513^a^
TSH level	1.56 ± 0.58	2.33 ± 0.88	3.591	0.001^a^^∗^

TSH: thyroid-stimulating hormone; HDRS: Hamilton Depression Rating Scale; MDD: major depression disorder; HC: healthy control; YMRS: Young Manic Rating Scale. ^a^Mann-Whitney *U* test, ^∗^*p* < 0.05 significant. ^b^Chi-square test.

**Table 2:** Comparison of CF testing results in the two groups.

	MDD group	HC group	*t* or *Z*	*p* value	*p*_adj_
Processing speed	46.41 ± 8.62	55.58 ± 7.22	-4.248	0.001^a^	0.001^∗^
Attention/vigilance	50.95 ± 9.92	56.25 ± 9.53	-2.004	0.051^a^	0.058
Working memory	45.60 ± 11.28	52.82 ± 9.36	-2.566	0.013^a^	0.021^∗^
Verbal learning	47.51 ± 11.52	53.64 ± 6.87	-2.302	0.025^a^	0.033^∗^
Visual learning	47.07 ± 10.83	56.22 ± 9.51	-3.309	0.002^a^	0.004^∗^
Reasoning problem-solving	49.19 ± 6.92	55.59 ± 7.86	-3.193	0.002^a^	0.004^∗^
Social cognition	47.52 ± 14.37	55.11 ± 12.15	-1.772	0.076^b^	0.076
Composite	46.55 ± 10.49	57.62 ± 7.54	-4.494	0.001^a^	0.001^∗^

CF: cognitive function; MDD: major depression disorder; HC: healthy control. ^a^*t*-test, *p* < 0.05 significant. ^b^Mann-Whitney *U* test, ^∗^*p* < 0.05 significant.

**Table 3:** Comparison of PFC and cerebellum biochemical metabolite ratios.

	MDD group	HC group	*t* or *Z*	*p* value	*p*_adj_
NAA/Cr of right PFC	1.82 ± 0.25	1.99 ± 0.33	-2.060	0.044^a^	0.117
Cho/Cr of right PFC	1.05 ± 0.19	1.13 ± 0.20	-1.557	0.125^a^	0.203
NAA/Cr of left PFC	2.05 ± 0.27	2.05 ± 0.34	-0.081	0.936^a^	0.936
Cho/Cr of left PFC	1.19 ± 0.18	1.23 ± 0.21	-0.833	0.409^a^	0.545
Right cerebellum NAA/Cr	1.11 ± 0.23	1.29 ± 0.21	-3.015	0.004^a^	0.032^∗^
Right cerebellum Cho/Cr	0.86 ± 0.34	0.93 ± 0.18	-2.386	0.017^b^	0.068
Left cerebellum NAA/Cr	1.08 ± 0.21	1.21 ± 0.25	-1.526	0.127^b^	0.203
Left cerebellum Cho/Cr	0.88 ± 0.18	0.89 ± 0.16	-0.708	0.479^b^	0.547

PFC: prefrontal cortex; MDD: major depression disorder; HC: healthy control; NAA/Cr: N-acetylaspartate/creatine; Cho/Cr: choline/creatine. ^a^*t*-test, ^∗^*p* < 0.05 significant. ^b^Mann-Whitney *U* test.

## Data Availability

The labeled dataset used to support the findings of this study are available from the corresponding author upon request.
